# Adenosquamous carcinoma of breast in a 19 years old woman: a case report

**DOI:** 10.1186/1477-7819-8-44

**Published:** 2010-05-27

**Authors:** Amit Agrawal, Shopon Saha, Ian O Ellis, Alache M Bello

**Affiliations:** 1 Professorial Unit of Surgery, City Hospital Campus, University of Nottingham, Nottingham University Hospitals, Nottingham, NG5 1PB, UK; 2 Department of Surgery, City Hospital Campus, Nottingham University Hospitals, Nottingham, NG5 1PB, UK; 3 Department of Histopathology, City Hospital Campus, University of Nottingham, Nottingham University Hospitals, Nottingham, NG5 1PB, UK; 4 Nottingham Breast Institute, City Hospital Campus, Nottingham University Hospitals, Nottingham, NG5 1PB, UK

## Abstract

**Background:**

Adenosquamous carcinoma of the breast is a rare form of metaplastic breast carcinoma. We report such a case in a 19 years old female.

**Case presentation:**

Case notes and histopathology were reviewed. Adenosquamous carcinoma was diagnosed on wide local excision and patient underwent skin-sparing mastectomy with Latissimus dorsi flap reconstruction.

**Conclusions:**

Adenosquamous carcinoma of the breast is a rare form of metaplastic breast carcinoma. Data on correct management, follow-up and prognosis are very limited but given the high potential for local recurrence, aggressive surgery may be the only option.

## Background

Adenosquamous carcinoma of the breast is a rare morphological variant of metaplastic breast carcinomas which was first described in early 1980s [1,2]. Since then several cases and case series have been reported. We present a case of adenosquamous carcinoma in a very young woman aged 19 years. We also present a mini-review of literature on this known but rare histological type of breast cancer.

## Case presentation

The 19 years old woman presented with approximately four weeks history of painless 5 × 5 cm breast lump/lumpiness. There was no history of trauma, nipple discharge or relation with menstrual cycle. There was no known family history of breast cancer. Palpation revealed a non-tender, mobile, hard left upper quadrant left breast lump. Mammogram was not performed given the very young age of the patient. Ultrasound revealed a prominent diffuse hypoechoic lesion. It appeared to be most likely benign lesion but as there was an element of uncertainty as to the nature of the lump on ultrasound, an ultrasound guided core biopsy (14G needle) was performed.

The core biopsy of the concerned area revealed parenchymal-rich breast tissue with columnar cell change and epithelial hyperplasia. In addition, there was fibroblastic stroma with focal elastosis containing an infiltrative adenosquamous proliferation. Diagnostic excision was recommended by the breast multi-disciplinary team (MDT) as the lesion appeared benign but with uncertain malignant potential.

**Figure 1:**
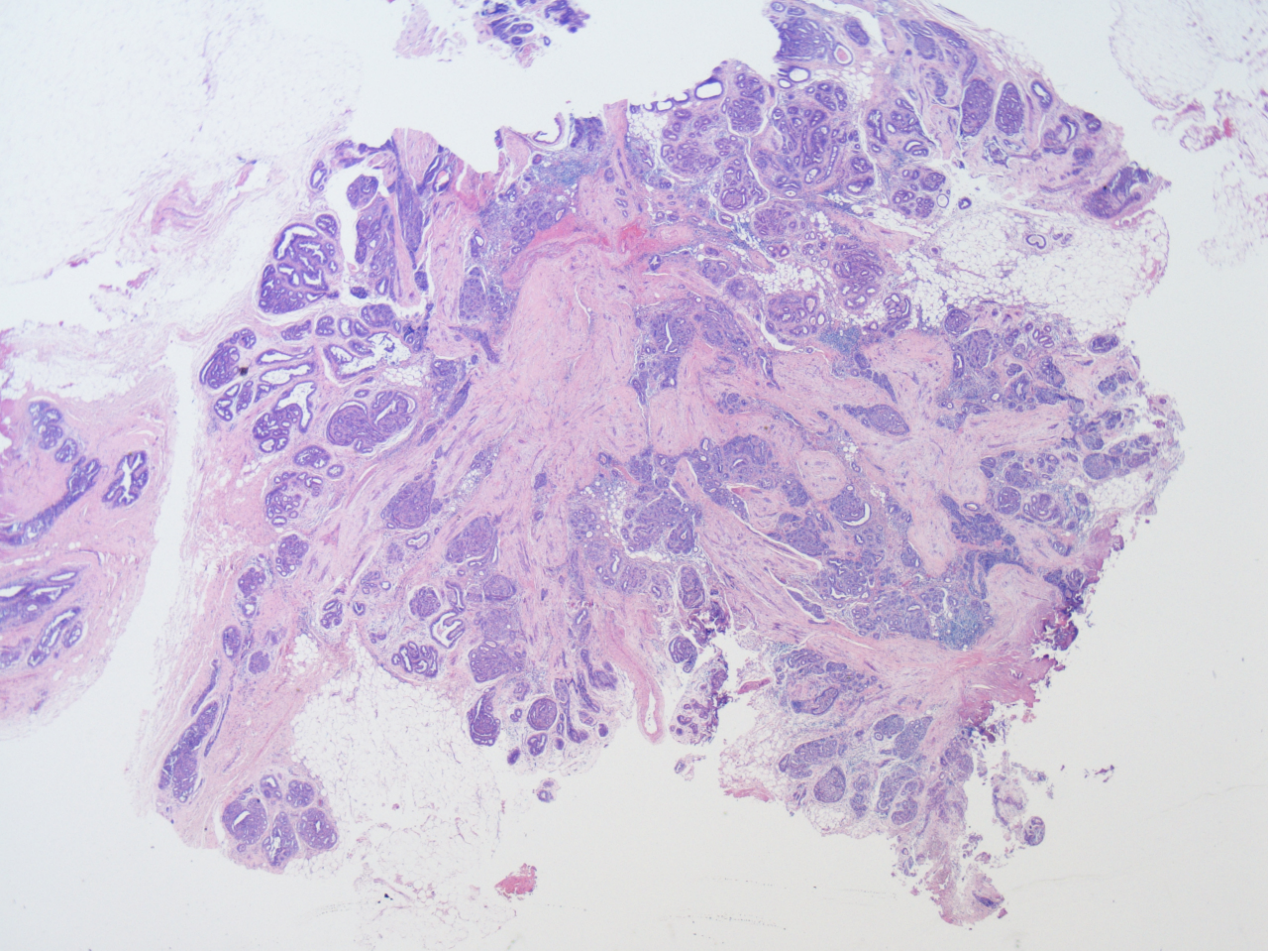
**Sclerosing lesion with central fibrosis and radiating parenchyma**. A low magnification (×20) view of the lesion. It has the characteristics of a sclerosing lesion with central fibrosis, entrapped parenchyma and radiating islands of tissue rich in parenchymal structures.

**Figure 2:**
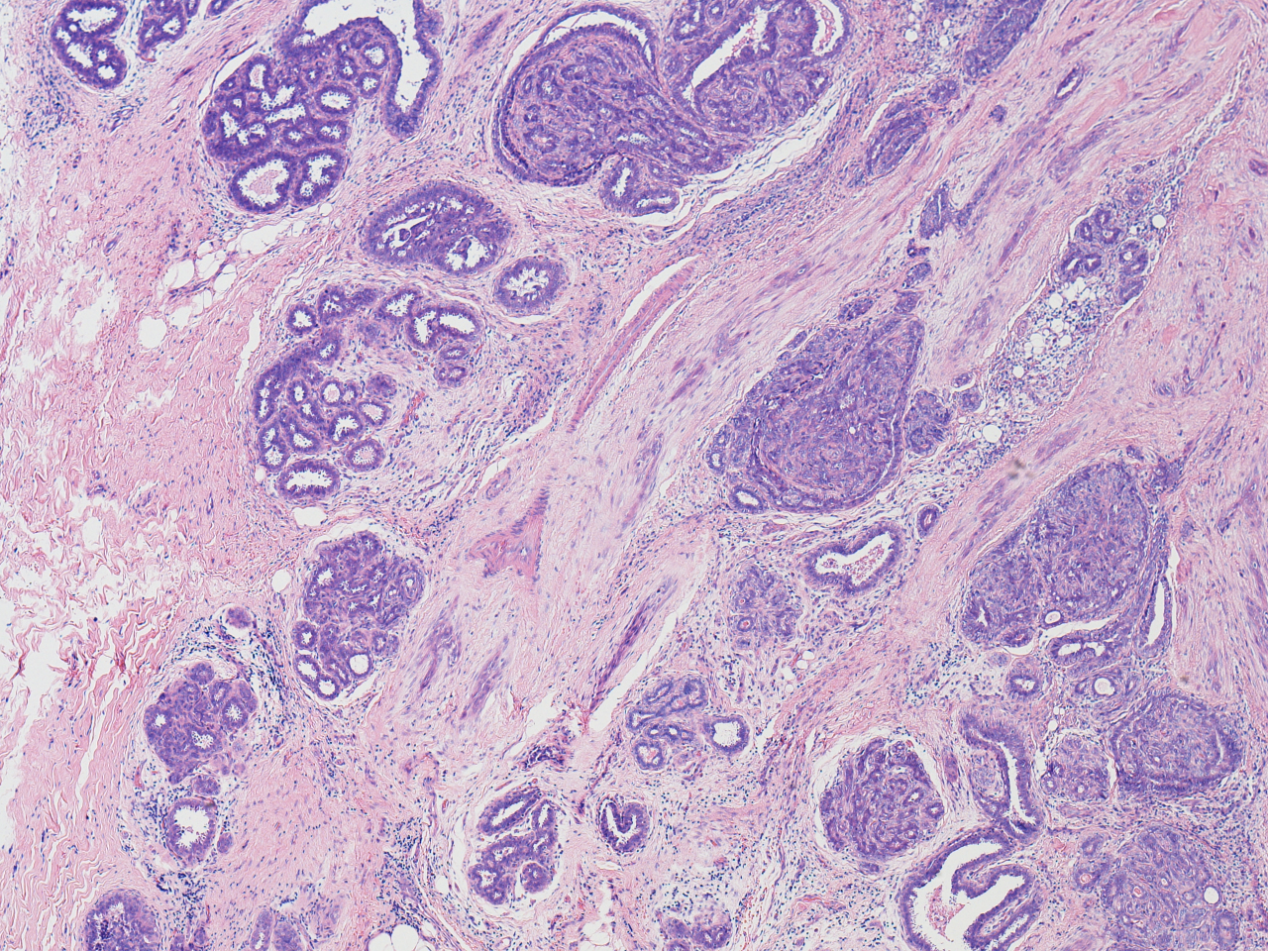
**View of one of the radiating arms of the lesion**. A view (×20) of one of the radiating arms of the lesion showing parenchymal tissue separated by a diagonal band of infiltrative cellular stromal tissue containing small ductal like structures.

**Figure 3:**
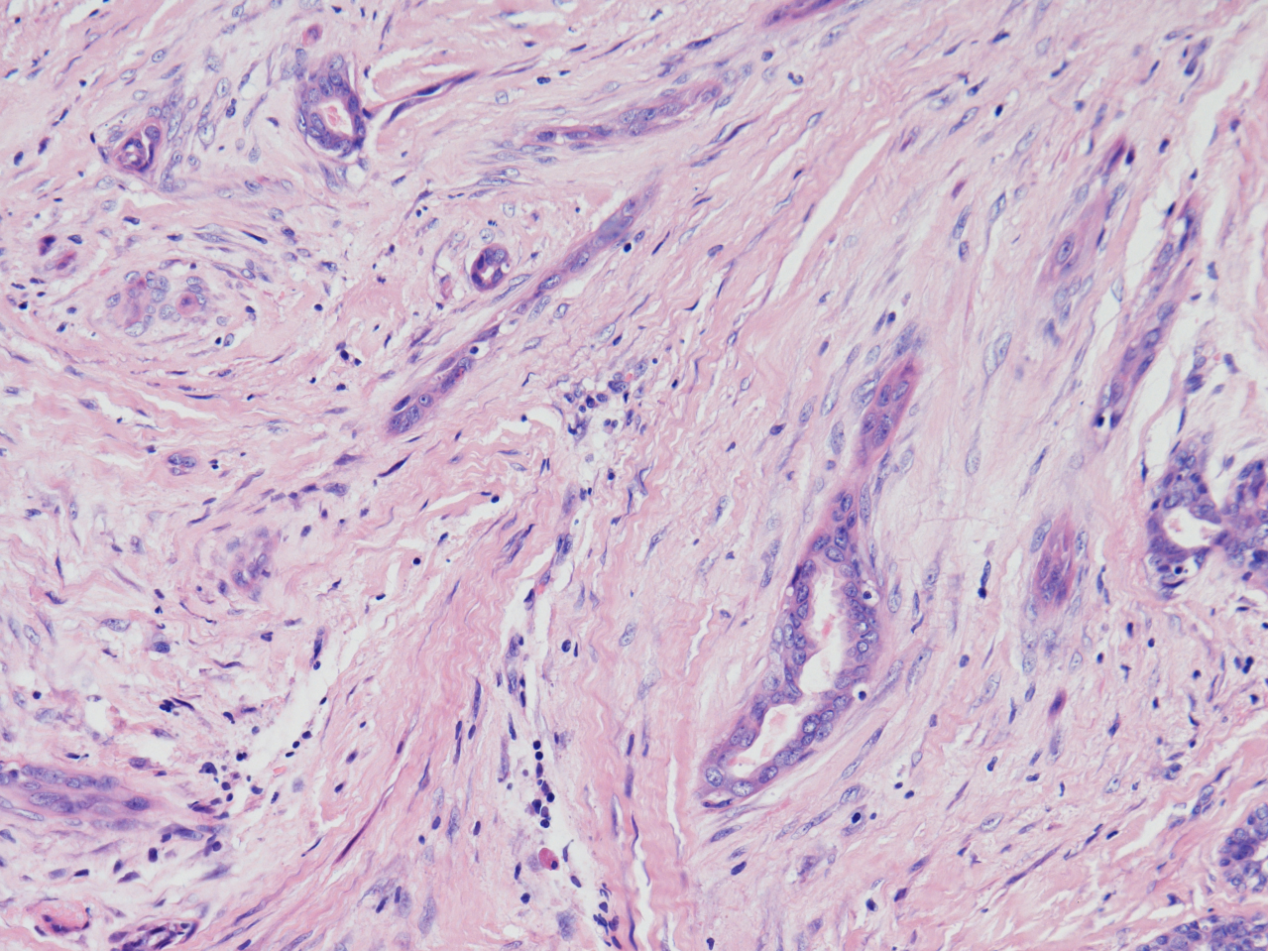
**Infiltrative but bland stromal tissue with ductal and epithelial structure**. A high magnification view (×40) of the infiltrative but bland cellular stromal tissue, along with the accompanying ductal and small epithelial structures. This combination of components is typical of low grade metaplastic adenocarcinoma.

As shown in Figures 1, 2 and 3, subsequent diagnostic excision biopsy showed an ill-defined area of benign adenosis set in fibroblastic stroma with focal elastosis suggestive of a radial scar or complex sclerosing lesion. Within this lesion there was an infiltrative proliferation of angulated glands showing adenosquamous features. A proportion of these glands lacked definite myoepithelial layers and the tumour expressed immunoreactivity to p63 gene antibody assay. Both the sclerosing lesion and the adenosquamous proliferation extended up to the excised margin. Hence, a wide local excision was deemed necessary.

The wide local excision specimen showed residual spindle cell lesion with squamoid islands seen in the infero-lateral shave specimen taken during wide local excision.

External opinion was sought from international experts (as acknowledged later) in this uncommon histological subtype of breast cancer. The consensus view was that it was low grade adenosquamous carcinoma.

Due to reported low potential for metastasis but high potential for local recurrence, she underwent Skin-sparing mastectomy with immediate Latissimus Dorsi flap reconstruction with an implant.

Following mastectomy, no further adjuvant therapy was deemed necessary due to the completeness of resection in the specimen histopathology and also due to the reported low metastatic potential and no known increased risk of contra-lateral disease. It was decided to simply follow-up patient clinically annually for five years due to known high risk of local recurrence and then to mammogram screening programme.

## Conclusions

Metaplastic carcinomas are known to be characterised by the presence of non-epithelial cellular elements [3]. The initial core biopsy of our case raised suspicion of adenosquamous carcinoma. However, due to the inherent limitations of the core biopsy, diagnostic excision was recommended by the histopathologist. Ho et al [4] have demonstrated in their small series of four cases that limited material in fine needle aspiration/core biopsy does not yield enough material for confirmatory histopathological diagnosis.

Though diagnostic morphological differentiation may not be possible in fine needle aspiration/core biopsy but immuno-expression of myoepithelial marker may be near-confirmatory. Reis-Filho et al [5] have reported in their series of 82 archival fine-needle aspirates that p63 marker was demonstrable in majority of such aspirates. The p63 gene (belonging to the larger p53 gene family) is expressed in the basal cells of a range of myoepithelial tissues including breast [5-8]. It is now known to be a sensitive and specific myoepithelial marker [9] and has been reported to be 86.7% sensitive and 99.4% specific for metaplastic carcinomas [8]. Adenosquamous breast carcinomas are known to be immuno-reactive to p63 antibody assays. The tumour is this case-report was immuno-reactive to p63 antibody.

There are data suggesting that it may carry a minimal risk of metastasis[10]. The exact nature of this tumour is controversial but it may represent a reactive process. Complex sclerosing lesion with an adenosquamous proliferation are of uncertain nature but have been reported to be associated together [11]. This tumour is thought to carry only a minimal risk for metastatic progression but it carries a significant risk for local recurrence [12]. Therefore, complete local excision or mastectomy to achieve clear margins appear appropriate in light of the fact that there is paucity of long-term follow-up data in these patients.

To conclude, adenosquamous carcinomas are a rare histopathological type of breast cancer. It should be considered in uncertain sclerosing lesions with an abundance of epithelial hyperplasia with signs of squamous differentiation in a predominant fibroblastic stroma. Immunoreactivity to p63 antibody appears to be near confirmatory of adenosquamous differentiation. Although it is not known to have high metastatic potential but it does have potential for local recurrence if not suitably excised. Therefore, aggressive local treatment is presently perceived to be the management of choice until large long-term follow-up data is available in literature.

## Consent

Written informed consent was obtained from the patient for publication of this case report and any accompanying images. A copy of the written consent is available for review by the Editor-in-Chief of this journal.

## Competing interests

The authors declare that they have no competing interests.

## Authors' contributions

AMB was clinically responsible for patient's care, conceptualised the study and revised the manuscript. IOE was responsible for pathological interpretation, images and revised the manuscript. AA and SS drafted the manuscript and revised it. All authors read and approved the final manuscript.
